# Rice Na^+^ absorption mediated by OsHKT2;1 affected Cs^+^ translocation from root to shoot under low K^+^ environments

**DOI:** 10.3389/fpls.2024.1477223

**Published:** 2024-10-15

**Authors:** Satomi Kanno, Shigeto Fujimura, Junko Takahashi, Chenyu Li, Takuro Shinano, Shin-ichi Nakamura, Nathalie Leonhardt, Jun Furukawa

**Affiliations:** ^1^ Institute for Advanced Research, Nagoya University, Nagoya, Japan; ^2^ Institute of Life and Environmental Sciences, University of Tsukuba, Ibaraki, Japan; ^3^ Aix Marseille University, French Alternative Energies and Atomic Energy Commission (CEA), National Center for Scientific Research (CNRS), Bioscience and Biotechnology Institute of Aix-Marseille (BIAM), Saint-Paul-lez-Durance, France; ^4^ Tohoku Agricultural Research Center, National Agriculture and Food Research Organization (NARO), Fukushima, Japan; ^5^ Center for Research in Radiation, Isotopes, and Earth System Sciences, University of Tsukuba, Ibaraki, Japan; ^6^ Graduate School of Science and Technology, Degree Programs in Life and Earth Sciences, University of Tsukuba, Ibaraki, Japan; ^7^ Research Faculty of Agriculture, Hokkaido University, Sapporo, Japan; ^8^ Faculty of Life Sciences, Tokyo University of Agriculture, Tokyo, Japan

**Keywords:** potassium, sodium, cesium, OsHKT2;1, transporter, *Oryza sativa* L., safe food production

## Abstract

^137^Cs diffused into the environment due to a nuclear power plant accident has caused serious problems for safe crop production. In plants, Cs^+^ is similar in its ionic form to K^+^. Cs^+^ is absorbed and transported mainly by the K^+^ transport mechanism. However, the full picture of the genes contributing to Cs^+^ transport and the transport mechanism of rice is still unclear. This study focused on OsHKT2;1, a candidate Cs^+^ transporter under low K^+^ conditions. To verify the ability of OsHKT2;1 to transport Cs^+^, the *OsHKT2;1* mutant (*hkt2;1*) was grown in a ^137^Cs-contaminated paddy field in Fukushima. The ^137^Cs concentration in *hkt2;1* aboveground was higher than in the wild type (WT), and the K concentration in these samples did not change between WT and *hkt2;1*, whereas the Na concentration was lower in *hkt2;1*. Uptake experiments with radioactive tracers (^22^Na^+^, ^43^K^+^, and ^137^Cs^+^) in hydroponic systems with different elemental compositions showed a negative correlation between Na^+^ and Cs^+^ accumulation in rice shoot cultivated under low K^+^ conditions. These results indicated that OsHKT2;1 does not directly contribute to Cs^+^ uptake but is an important factor in regulating Cs^+^ translocation by controlling Na^+^ accumulation. This indicates the possibility of controlling rice Cs content by regulating the Na^+^ environment during cultivation.

## 1 Introduction

K is a macronutrient for plants. K^+^ is present in the cytoplasm as an inorganic cation and is involved in basic cellular functions such as electrical neutralization with anions, regulation of cell membrane polarization, and osmotic regulation (Schroeder and Fang, 1991). Therefore, plant growth requires the uptake of a sufficient K amount from the environment and its proper transport to the whole plant cell. Plants have multiple transporter proteins and use these differently to control their transport. K^+^ transporters in plant cell membranes are broadly classified into three categories: shaker K^+^ channel family, HAK/KUP/KT K^+^ transporter family, and HKT transporter family (Véry and Sentenac, 2003; Véry et al., 2014). However, alkali metal ions, Na^+^ and Cs^+^, may be absorbed and transported via these K^+^ transporters due to their similarity in chemical properties as ions (Amtmann and Sanders, 1998; Blumwald, 2000; Hampton et al., 2004; Adams et al., 2013). The permeability of transporters to more than one ion has been the focus of attention due to the problem of radioactive Cs^+^ diffused into agricultural lands after the accident at the TEPCO Fukushima nuclear power plant in 2011. Because Cs^+^ uptake by plants is enhanced under low K^+^ conditions, the effectiveness of K fertilization in suppressing Cs^+^ uptake has become widely known in general and has been used as a real countermeasure (Fujimura et al., 2014; Kato et al., 2015; Kubo et al., 2015; Matsunami et al., 2021). Indeed, most K^+^ transporters that function under low K^+^ conditions also have a permeability to Cs^+^. This is confirmed by the high contribution of OsHAK1 in rice Cs^+^ uptake (Nieves-Cordones et al., 2017; Rai et al., 2017). Studies have reported the contribution of AtHAK5 in Cs^+^ transport in *Arabidopsis thaliana* (Qi et al., 2008), and some members of cyclic nucleotide-gated channels are involved in Cs^+^ uptake (Kanter et al., 2010; Leng et al., 2002; Rai and Kawabata, 2020). However, because Cs^+^ uptake still exists even when the abovementioned genes are deleted, other genes are responsible for Cs^+^ transport. Among K^+^ transport genes that function under low K^+^ conditions, this study focused on OsHKT2;1, whose Cs^+^ transport activity has been confirmed by electrophysiology experiments using heterologous cells, *Xenopus laevis* oocytes (Jabnoune et al., 2009), but not in intact rice plants.

HKTs belong to the Trk/Ktr/HKT superfamily of K^+^ transporters, are conserved in microbes and plants (Hamamoto et al., 2015), and are classified into two classes in plants. Class 1 (HKT1s) is a Na^+^-selective transporter (Garciadeblás et al., 2003), and class 2 (HKT2) has been isolated as a K^+^ transporter and confirmed to be a Na^+^ and K^+^ cotransporter (Oomen et al., 2012; Suzuki et al., 2016a). Eight *HKT* genes have been reported in rice, four belonging to class 1 (OsHKT1;1, OsHKT1;3, OsHKT1;4, and OsHKT1;5) and four to class 2 (OsHKT2;1, OsHKT2;2, OsHKT2;3, and OsHKT2;4). HKT is an important factor in salt tolerance due to its ability to transport Na^+^ (Véry et al., 2014). OsHKT1;1 OsHKT1;4, and OsHKT1;5 prevent Na^+^ overaccumulation in shoots by ensuring Na^+^ exclusion from the xylem (Hauser and Horie, 2010; Khan et al., 2020). OsHKT2;1 has a lower K^+^ permeability than other class 2 transporters and shows a strong preference for Na^+^ due to the amino acid sequence in the first region of the p-loop (Horie et al., 2001). Therefore, it is mainly involved in root Na^+^ uptake in rice (Horie et al., 2001, 2007; Golldack et al., 2002; Garciadeblás et al., 2003; Miyamoto et al., 2015). In contrast, OsHKT2;2, OsHKT2;3, and OsHKT2;4 exhibit complicated ion selectivity among Na^+^, K^+^, Mg^2+^, and Ca^2+^ in heterologous expression experiments (Horie et al., 2001, 2011; Lan et al., 2010). Na^+^ ions absorbed by plants often cause salt stress and reduce crop yields; therefore, many studies related to Na^+^ stress tolerance have been reported. However, Na^+^ ions are present in plants at trace levels (10 mg kg^−1^–1,200 mg kg^−1^); in some species, Na^+^ is considered a beneficial element (some C_4_ and CAM plants need Na^+^ as an essential element). In particular, rice takes up Na^+^ under low K^+^ conditions; Na^+^ uptake as a K^+^ replacement recovers biomass loss due to K^+^ deficiency, and OsHKT2;1 is involved in alleviating K^+^ deficiency by Na^+^ absorption (Horie et al., 2007; Ochiai et al., 2022).

Further studies have shown that HKT2;1 functions as a Na^+^ transporter under low K^+^ and high Na^+^ conditions and as a Na^+^ and K^+^ cotransporter under low K^+^ and low Na^+^ conditions (Jabnoune et al., 2009; Yao et al., 2010). In addition to Na^+^ and K^+^ transport activities, Jabnoune et al. identified Cs^+^ transport activity of OsHKT2;1 with the electrophysiology experiments (Jabnoune et al., 2009). Therefore, the ion selectivity of OsHKT2;1 among Na^+^, K^+^, and Cs^+^, should depend on the soil conditions being cultivated. To investigate OsHKT2;1 contribution to the accumulation of these monovalent cations in a real paddy field, this study performed field cultivation experiments in radioactive Cs-contaminated soil using OsHKT2;1 knockout (KO) rice plants. In addition, based on the Na^+^ and K^+^ concentrations of cultivating soil solution, detailed ion uptake and translocation were verified with hydroponics reproducing the paddy field environments. Combining these two experiments, this study identified low Na accumulation in OsHKT2;1 KO rice and Na-induced suppression of Cs translocation from roots to shoots. This phenomenon might be a countermeasure for preventing ^137^Cs accumulation in rice shoots and grains.

## 2 Materials and methods

### 2.1 Plant materials and growth conditions

Two Tos-17 lines of OsHKT2;1 (NC2534 and ND4057) used in this study were selected by BLAST searches against a dataset of Tos-17 flanking sequences in the rice genome (Miyao et al., 2003). In both lines, Tos-17 insertions were identified and OsHKT2;1 mutant line (*hkt2;1*) and Tos-17 escaped WT one were established. Seeds were sterilized and germinated in water at 30°C for 3 days in the dark. Seedlings were grown under 16-h light/8-h dark cycles at 28°C in the nutrient solution [0.5 mM (NH_4_)_2_SO_4_, 1.6 mM MgSO_4_·7H_2_O, 1.55 mM Ca(NO_3_)_2_·4H_2_O, 0.4 mM H_3_PO_4_, 0.1 mM Fe-EDTA(Na-free), 10 µM MnSO_4_·H_2_O, 0.162 µM (NH_4_)_6_Mo_7_O_24_·4H_2_O, 0.7 µM ZnSO_4_·7H_2_O, 0.8 µM CuSO_4_·5H_2_O, and 22.6 µM H_3_BO_3_, adjust to pH 5.5 with Tris–HCl] with several KCl and NaCl concentrations. For the experiment to reproduce ion concentrations in the paddy field, plants were grown in the above solution for 12 days, with several Na^+^ and K^+^ concentrations based on the measurements in June, just after rice transplanting to the paddy field (20 µM K^+^ and 800 µM Na^+^ for low K^+^ and 40 µM K^+^ and 800 µM Na^+^ for high K^+^) or August, around rice heading date (20 µM K^+^ and 300 µM Na^+^ for low K^+^ and 30 µM K^+^ and 400 µM Na^+^ for high K^+^). For the radioisotope tracer uptake experiment with several Na^+^ concentrations, plants grown with 100 µM K^+^ for 7 days and transferred to 10 µM K^+^ for 5 days were treated with the radioisotope containing solution for 2 days (**Supplementary Figure 1A**).

### 2.2 Rice growth experiments in the ^137^Cs-contaminated paddy field

For experiment 2019, seeds were sterilized and germinated in water at 30°C for 3 days in the dark. Seedlings were grown under 16-h light/8-h dark cycles at 28°C in hydroponic cultures with 1/2 Kimura B solution for 20 days (Kimura, 1931). For experiments 2020 and 2021, seeds were sown in the soil directory and grown for 21 days, 34 days each, in the greenhouse. Fertilizer (N: ammonium sulfate 2.1 kg/10 a, coated urea 4.2 kg/10 a; and P: superphosphate lime 2.6 kg/10 a) was plowed into the soil before the fields were filled with water. The paddy field was divided into two plots: one with additional K fertilizer (K: 10 g/m^2^) and the other without. Seedlings were planted every 30 cm in the paddy field and grown for 5 months.

### 2.3 ^137^Cs measurement by a germanium counter

The aerial parts of rice were taken as samples. Four to six individual plants were assembled to form one sample, and four samples were taken from each line. After drying, the leaves and stems, husk, and brown rice were separated, and the ^137^Cs amount was measured using a germanium counter (GC2520-7500SL, GC4020-7500SL, GCW2523-7905-30U-ULB, Mirion Technologies, Inc.). Roots were not used as samples due to the unavoidable ^137^Cs contamination of the soil.

### 2.4 Na and K measurement in plant samples using an atomic absorption photometer

Rice shoots were dried for 2 days at 80°C. Dried samples were digested in 60% HNO_3_ at 100°C for 1 h. Acid solutions were diluted with water and filtrated. Na and K concentrations in the solution were determined by an atomic absorption photometer (ZA3000, Hitachi High-Technologies).

### 2.5 Na^+^ and K^+^ measurement in aqueous field solution by ion chromatography

Soil solutions were collected in June and August from different K fertilization zones by centrifugation of fresh soil according to Yamasaki and Kishita (1972). A soil solution expected to be present in the soil at pF 4.2 was collected by centrifugation at 8,700 rpm for 60 min using a high-speed refrigerated centrifuge (SS-2050A, Sakuma Seisakusho) and a rotor dedicated to soil (HB-RL, Sakuma Seisakusho). The collected soil solutions were filtered with a 0.20-μm filter, and the concentrations of Na^+^ and K^+^ were quantified using ion chromatography (Prominence, Shimadzu).

### 2.6 Radioisotope tracer (^22^Na^+^, ^43^K^+^, and ^137^Cs^+^) uptake experiment

For the experiment to reproduce ion concentrations in the paddy field, plants grown as described in Section 2.1 were treated for 2 days with four conditions (June high K^+^/low K^+^ or August high K^+^/low K^+^) with 0.01 mM CsCl and ^22^Na^+^ (0.03 kBq/ml), ^43^K^+^ (0.5 kBq/ml), or ^137^Cs^+^ (0.03 kBq/ml). For investigating Na^+^ dose response, plants grown as described in Section 2.1 were treated for 2 days with 0.01 mM, 0.5 mM, 1 mM, 5 mM, 10 mM, and 50 mM NaCl with ^22^Na^+^ (0.03 kBq/ml), 0.01 mM KCl with ^43^K^+^ (0.5 kBq/ml), or 0.01 mM CsCl with ^137^Cs^+^ (0.03 kBq/ml). The samples were separated into shoots and roots and weighed, and γ-radiation was measured with a γ-counter (AccFLEX γ7001, Aloka). The amount of total (including radioactive and non-radioactive) Na, K, and Cs was calculated based on the radioactivity and concentration of each solution.

### 2.7 RNA sequencing analysis

Plants grown as described in Section 2.1 were treated for 1 day with several conditions that showed differences in ^137^Cs^+^ translocation from roots to shoots. Total RNA was extracted from 18 samples (n = 3, biological replicates), divided into shoot and root sections, for WT (Na^+^ 100 µM treatment), WT (Na^+^ 10 mM treatment), and NC2534 type *hkt2;1* (Na^+^ 10 mM treatment) plants. The RNA library was prepared at the NODAI Genome Research Center using Illumina’s NEB Next Ultra II Directional RNA Library Prep Kit for Illumina (New England Biolabs) and analyzed using NextSeq 1000 (Illumina), paired end read 2 × 150 bp. Data were extracted for differentially expressed genes (DEGs) with fold change ≥ |2| and false discovery rate (FDR) adjusted *p* < 0.05. Two comparative analyses were performed [WT Na^+^ 100 µM treated vs. WT Na^+^ 10 mM treated and *hkt2;1* Na^+^ 10 mM treated vs. WT Na^+^ 10 mM treated].

### 2.8 Gene ontology enrichment analysis

Gene ontology (GO) enrichment analysis was performed for DEGs in each comparison group using AgriGO version 2.0 (Tian et al., 2017). Selected singular enrichment analysis was performed using Fisher’s statistical test method and Yekutieli’s method (FDR under dependency). The significance level was set as 0.05.

### 2.9 RNA extraction and RT-qPCR analysis

Samples grown hydroponically were collected separately from roots and aerial parts, frozen in liquid nitrogen, and ground. Total RNA was extracted from crushed samples using Direct-zol RNA Miniprep (Zymo Research). cDNA was synthesized using qScript cDNA SuperMix (Quanta). RT-qPCR was performed with LightCycler 480 SYBR Green I Master PCR Master (Roche) on a LightCycler 480 (Roche) according to the manufacturer’s protocols. Amplification reactions were performed in a total volume of 5 μl, which contained 2 μl cDNA, 2.5 μl SYBR Green premix, and 0.5 μl forward and reverse primers (1 μM). PCR was programmed as follows: 90°C for 10 min, followed by 40 cycles of 95°C for 15 s and 60°C for 1 min. PCR efficiency was 100% for each pair of primers, and a threshold value was determined. The specificity of PCR amplification was examined by monitoring the presence of the single peak in the melting curves after RT-qPCR. Relative gene expression in each sample was compared to the control and calculated using the ΔΔCt method. Using this analysis, the relative gene expression in the control sample was equal to 1, and the relative expression of the other treatments was compared to control plants. The housekeeping genes were actin (*Os03g0718100*) and ubiquitin (*Os01g0328400*). Subsequent RT-qPCR was performed in triplicate for each sample. Primer sequences are provided in **Supplementary Table 1**.

### 2.10 Statistical analysis

Two-way analysis of variance (ANOVA) and Tukey’s multiple comparison were performed using GraphPad Prism version 8.00 for MacOS X (GraphPad Software, La Jolla, CA, USA; www.graphpad.com).

## 3 Results

### 3.1 Rice growth in the ^137^Cs-contaminated paddy field demonstrated that OsHKT2;1 does not contribute directly to ^137^Cs^+^ transport

**Figure 1:**
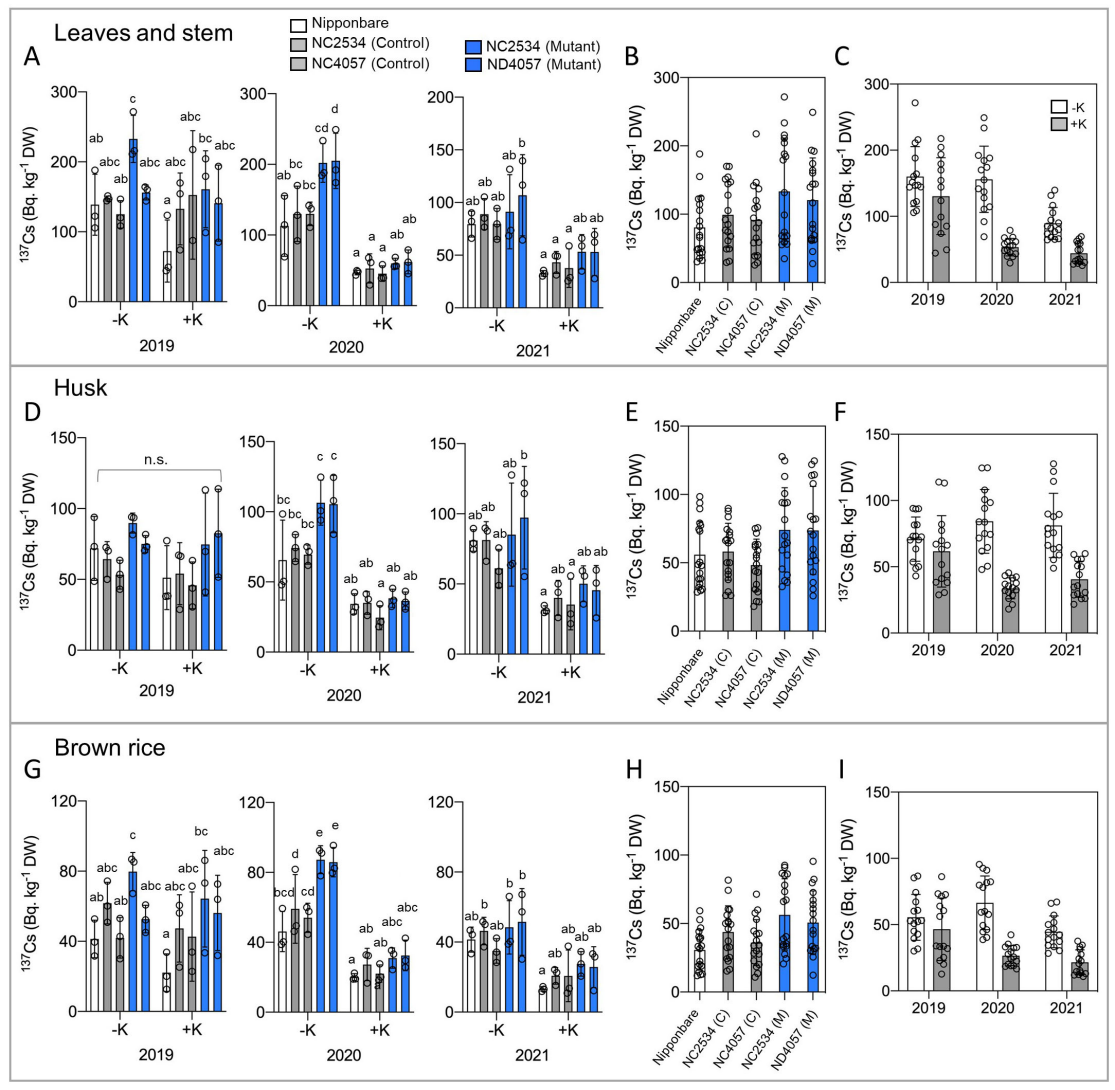
^137^Cs concentrations of wild type and *hkt2;1* grown in the paddy field of Fukushima. **(A, D, G)** ^137^Cs concentrations of leaves and stem, husk, and brown rice of sample grown in paddy field contaminated with ^137^Cs for ~5 months. From left to right: Nipponbare (white), WT of NC2534, WT of ND4057 (gray), mutant type NC2534, and mutant type ND4057 (blue). Data are mean ± SD. n = 3 (biologically independent experiments) and individual data points as overlays. **(B, E, H)** Comparison of the full sample of 3 years of data in each line without considering K treatment. **(C, F, I)** Differences between with and without K fertilizer application in each year without considering lines. Different letters in **(A, D, G)** indicate statistically significant differences (*p* < 0.05, two-way ANOVA, Tukey’s multiple comparisons). Because of the detailed comparison shown in **(A, D, G)**, no multiple comparisons between lines **(B, E, H)** and years **(C, F, I)** were added.

To investigate the effects of environmental ^137^Cs on agricultural rice cultivation, this study aimed to elucidate the phenomena observed in actual paddy fields. The laboratory environment often deviates from the actual environment; therefore, a rice cultivation experiment was first conducted in an experimental field that had been preserved without soil conditioner (Zeolite) after the nuclear power plant accident. Three field experiments were conducted between 2019 and 2021. The field was divided into two plots, and K fertilizer (K: 10 g/m^2^) was applied to one of the soils after irrigation. Each year, seedlings were transplanted to the field between May and June and grown for ~5 months with conventional irrigation. The samples were separated aboveground into leaves and stems, husks, and brown rice to determine the ^137^Cs content (**Figures 1A, D, G**). Roots were not measured as it was not possible to prevent ^137^Cs containing soil contamination.

The data obtained showed the tendency that ^137^Cs accumulation was suppressed in K fertilizer treatment in all investigated organs in 2020 both in WT and mutants, whereas the significance was decreased or not observed in 2021 and 2019, respectively (**Figures 1A, C, D, F, G, I**). Under the no K fertilizer condition, ^137^Cs accumulation was higher in the HKT2;1 mutant in all organs in 2020 (**Figures 1A, D, G**). Focusing on each year, significant differences were obtained in 2020 and in some comparisons in 2021 of the three experiments; there were no significant differences in 2019, possibly due to seedling preparation or irrigation or weather conditions. In the consideration of HKT2;1 mutation, ^137^Cs accumulation was higher in husk and brown rice of mutant plants than WT plants (**Figures 1E, H**) and leaves and stems showed no significant difference between lines (**Figure 1B**). However, even in leaves and stems, 2019 and 2020 data showed higher ^137^Cs accumulation in mutant (**Figure 1A**). These results were contrary to the initial prediction that OsHKT2;1 could transport Cs^+^. If OsHKT2;1 contributed directly to Cs**^+^** transport, Cs**^+^** uptake should be lower in the *hkt2;1*, but the results indicated ^137^Cs in *hkt2;1* was higher than in WT, suggesting that OsHKT2;1 does not contribute to direct Cs**^+^** transport in field-grown rice plants.

### 3.2 K and Na content in WT and mutants in paddy field experiments

**Figure 2:**
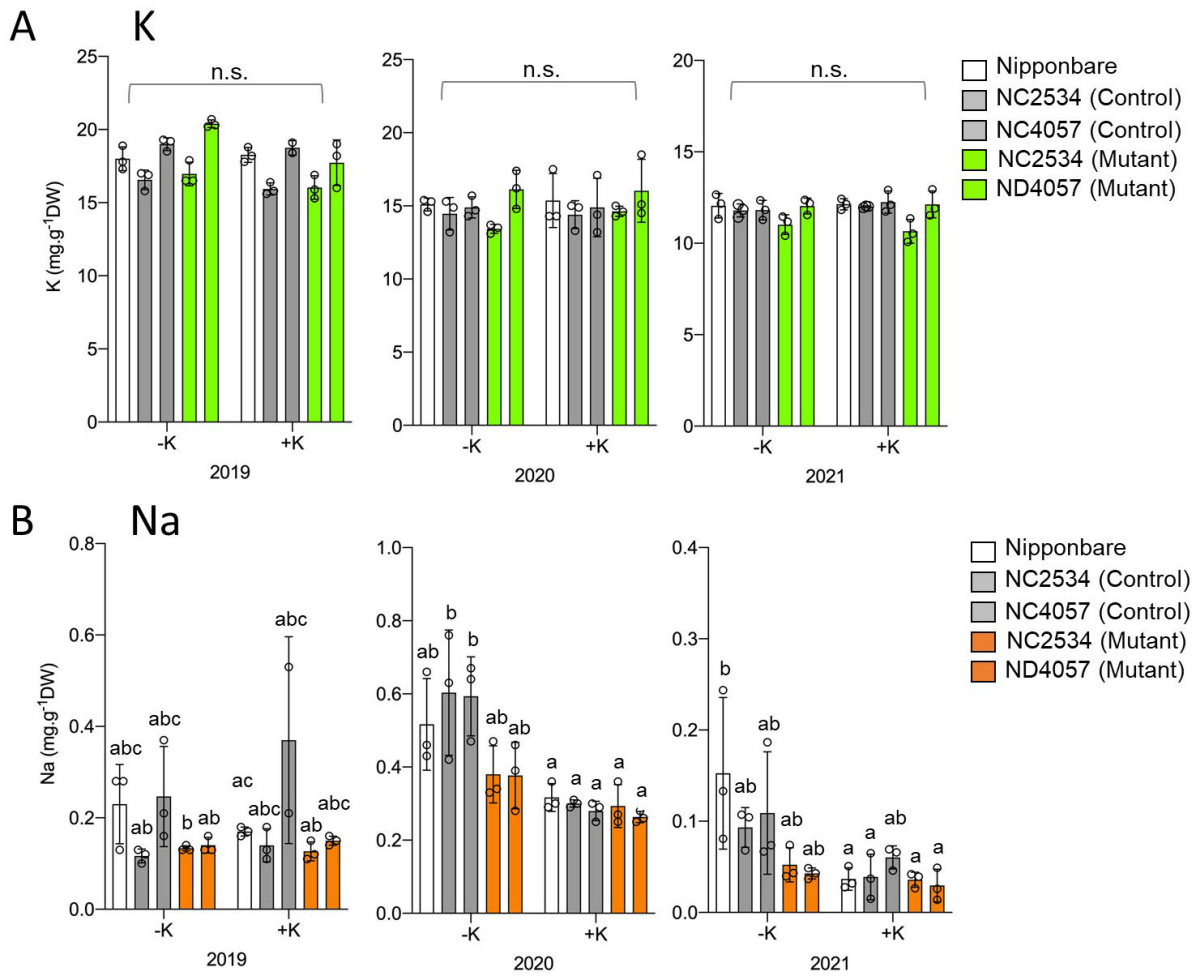
Comparison of K and Na concentrations between wild type and *hkt2;1* grown in the paddy field of Fukushima. **(A)** K concentration of leaves and stem samples grown for ~5 months in the ^137^Cs-contaminated paddy field. From left to right: Nipponbare (white), WT of NC2534, WT of ND4057 (gray), mutant type NC2534, and mutant type ND4057 (green). **(B)** Na concentration of leaves and stem samples same as **(A)** From left to right: Nipponbare (white), WT of NC2534, WT of ND4057 (gray), mutant type NC2534, and mutant type ND4057 (orange). Data are the mean ± SD. n = 3 (3 times biologically independent experiments) and individual data points as overlays. Different letters indicate statistically significant differences (*p* < 0.05, Two-way ANOVA, Tukey’s multiple comparisons). n.s. means not significant.

HKT2;1 is a Na^+^ and K^+^ cotransporter; therefore, it was speculated that the higher ^137^Cs content in the mutants might be the effect of reduced K^+^ uptake due to the loss of OsHKT2;1. Therefore, after ^137^Cs measurement, the samples were digested with nitric acid and K concentration was quantified, but no difference was observed between WT and mutants both under +K and -K conditions (**Figure 2A**). In contrast to K concentration, the Na concentration was significantly higher under no K fertilizer condition and decreased in mutant plants in 2020, whereas the significance was decreased or not observed in 2021 and 2019, respectively (**Figure 2B**).

### 3.3 Reproduction of shoot Cs accumulation with laboratory hydroponics system

**Figure 3:**
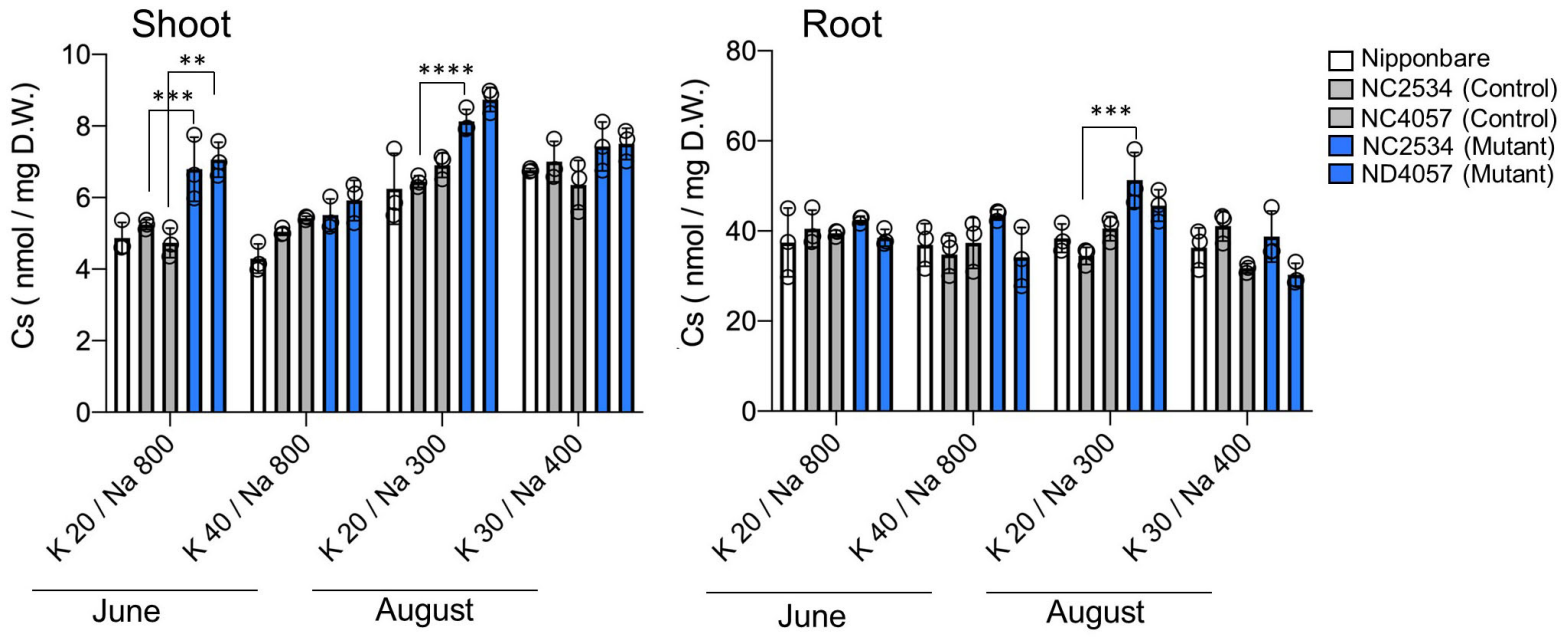
Cs absorption with hydroponic solutions reproducing Na^+^ and K^+^ concentrations in the paddy field. From left to right: Nipponbare (white), WT of NC2534, WT of ND4057 (gray), mutant type NC2534, and mutant type ND4057 (blue). Data are the mean ± SD (n = 3, three times biologically independent experiments) and individual data points as overlays. (**p = 0.0011, ***p < 0.0003, ****p < 0.0001, Two-way ANOVA, Tukey’s multiple comparisons).

Based on field experiments, there was an obvious negative relationship between the Na and ^137^Cs concentrations in the plants treated without K fertilizer in 2020 (**Figures 1A**, **2B**). Therefore, the details of the effect of Na on Cs accumulation were examined under hydroponic experimental conditions in the laboratory. To establish the hydroponic conditions, the concentrations of the K**^+^** and Na**^+^** in the irrigation water and soil solution of the experimental field was focused as a reference. The K**^+^** and Na**^+^** concentrations in the supernatant solution of aqueous soil solutions collected randomly from several locations in the field were measured (**Table 1**). Results obtained just after transplanting the seedlings (June) showed Na**^+^** 759 ± 225 µM (mean ± SD) and K**^+^** 45 ± 25 µM in the K fertilized area and Na**^+^** 771 ± 87 µM and K**^+^** 20 ± 16 µM in the K non-fertilized area. Two months after transplanting and around the rice heading date (August), results showed Na**^+^** 393 ± 27 µM and K**^+^** 27 ± 4 µM in the K fertilized plot and Na**^+^** 295 ± 47 µM and K**^+^** 21 ± 3 µM in the K non-fertilized plot, indicating approximately 1.3-fold difference in K**^+^** concentration between with and without K fertilization and that Na**^+^** was reduced by approximately half its initial concentration over the 2-month period. The hydroponic solution was adjusted based on the above concentrations, and different tracers ^22^Na^+^, ^43^K^+^, and ^137^Cs^+^ were added to hydroponic solutions subjecting to the uptake experiments. As a result, total Cs accumulation calculated by the ^137^Cs radioactivity in shoots increased in both mutants at K**^+^** 20 µM/Na**^+^** 800 µM (−K condition in June), confirming the reproduction of the field experiments with the laboratory hydroponic experimental system (**Figure 3**). In the K accumulation, high K concentrations in shoot and root were observed under Na**^+^** 800-µM conditions in all lines; however, the difference between WT and mutants was not observed (**Supplementary Figure 2**). Different from K accumulation, suppression of Na uptake was clearly shown in HKT2;1 mutant under Na**^+^** 800 µM conditions, suggesting that HKT2;1 contributed to the large part of Na^+^ uptake under these conditions. Based on the shoot and root concentrations of Cs and Na in each sample shown in **Figure 3** and **Supplementary Figure 2**, the relationships among shoot Cs, root Cs, shoot Na, and root Na concentrations were investigated. Instead of no obvious relationship in root Cs and Na, there is a negative relationship between shoot Cs and Na (**Supplementary Figure 3**). The obtained R^2^ value revealed that the negative relationship was well observed between “Shoot Cs” and “Root Na” comparing to others (**Supplementary Table 2**).

**Table 1:** K^+^ and Na^+^ concentrations of soil solution in the paddy field.

	June (just after tranplanting)		August (around headings)	
	Na^+^ (μM)	K^+^ (μM)	Na^+^ (μM)	K^+^ (μM)
Soil solution (K+)	759 ± 225	45 ± 25	393 ± 27	27 ± 4
Soil solution(K-)	771 ± 87	20 ± 16	295 ± 47	21 ± 3
Flooded water	334 ± 19	32 ± 3	114 ± 12	44 ± 9

The K^+^ and Na^+^ concentrations in aqueous solutions randomly collected from six locations in the plot of paddy field were measured. Soil solution was collected at two different times, just after transplanting the seedlings (June) and around rice heading date (August). Mean ± SD (n = 3–5).

### 3.4 Comparison of Na^+^, K^+^, and Cs^+^ uptake in different Na^+^ concentrations by hydroponic experimental conditions

**Figure 4:**
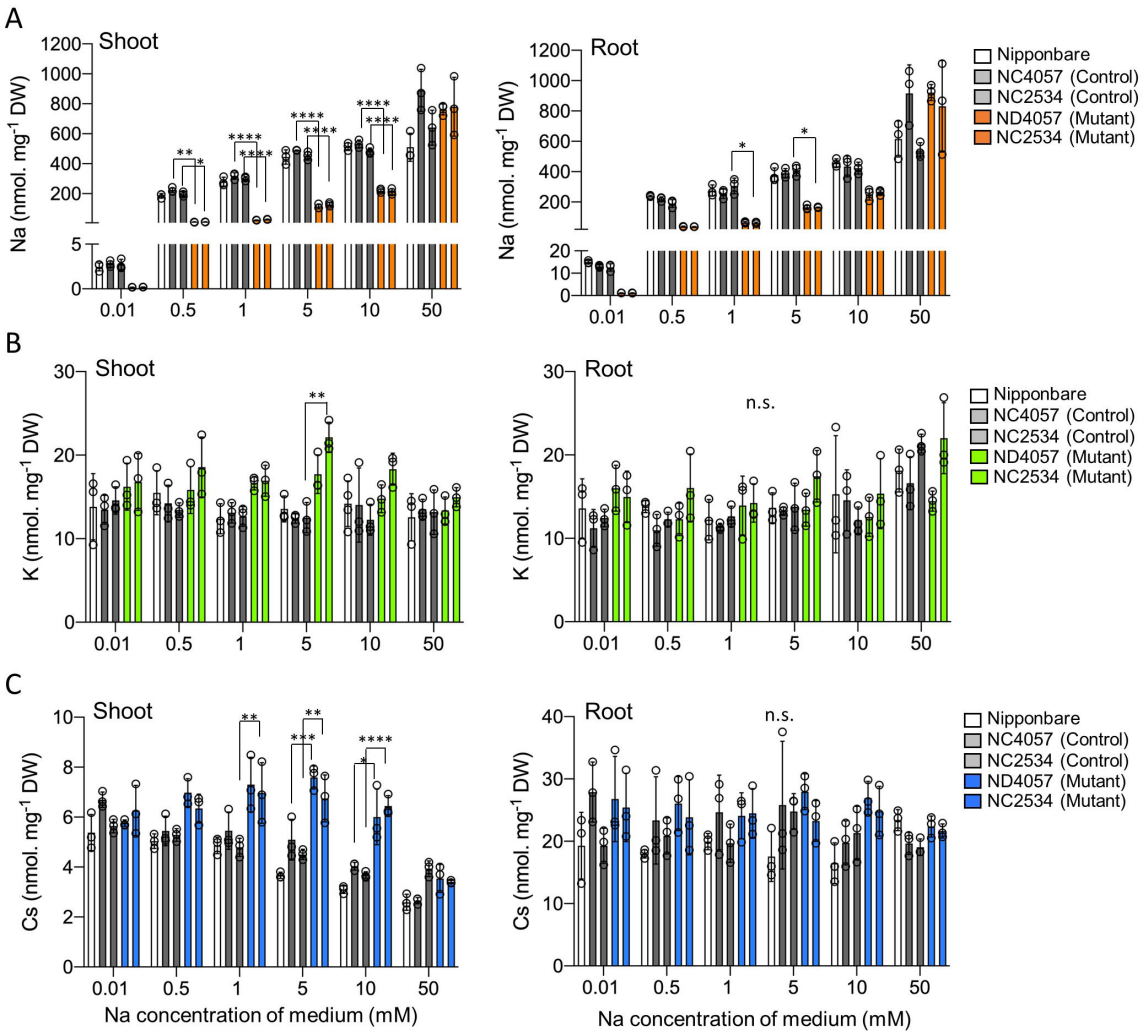
Comparison of Na, K, and Cs accumulation with different Na^+^ treatments. **(A)** Na concentration in shoot and root after 48 h treatment. From left to right: Nipponbare (white), WT of NC2534, WT of ND4057 (gray), mutant type NC2534, and mutant type ND4057 (orange). **(B)** K concentration of shoot and root samples same as **(A, C)**. Cs concentration of shoot and root samples same as **(A)**. Data are the mean ± SD (n = 3, three times biologically independent experiments) and individual data points as overlays. (*p < 0.05; **p < 0.005; ***p < 0.0002; ****p < 0.0001, two-way ANOVA, Tukey’s multiple comparisons). n.s. means not significant.

Based on the results of reproduction of the field experiment with the laboratory hydroponics, this study proceeded with the next experiment with K^+^ concentrations around <20 µM. The samples were treated with 100 µM K**^+^** for 7 days and 10 µM K**^+^** for 5 days before the uptake experiments. It was confirmed that *OsHKT2;1* expression was induced by 10 µM K**^+^** for 5 days in WT plants (**Supplementary Figure 1**). For the uptake experiments, different tracers ^22^Na^+^, ^43^K^+^, and ^137^Cs^+^ were added to hydroponic solutions at different Na^+^ concentrations ranging from 0.01 mM to 50 mM for 2 days. Non-radioactive Na, K, and Cs concentrations absorbed were calculated by the ratio of the initial concentrations. Na concentrations increased in both WT and mutants according to the applied Na^+^ throughout investigated Na^+^ ranges. However, from 0.01 mM to 10 mM, the Na concentration was lower in mutants than in WT, and no significant difference at 50 mM (**Figure 4A**), indicating that OsHKT2;1 contributed significantly to Na^+^ uptake at 0.01 mM to 10 mM. K uptake and translocation were independent of the Na^+^ concentration in the solution (**Figure 4B**). With 5-mM Na^+^ treatment, shoot K accumulation was higher in one mutant than in its WT, but no significant difference was observed in another mutant. Regarding Cs accumulation, both *hkt2;1* lines, which suppressed Na^+^ uptake, showed higher Cs accumulation in shoots compared to WT at the 5-mM to 10-mM Na^+^ treatment (**Figure 4C**). However, no such difference was observed in roots. Interestingly, WT showed a significant decrease in Cs in shoots according to the applied Na^+^ concentration; however, no similar trend was observed in roots. These data supported the existence of a negative correlation between Na^+^ and Cs^+^ concentrations in shoot.

### 3.5 RNA-seq analysis to identify genes that fluctuate with Cs translocation

**Figure 5:**
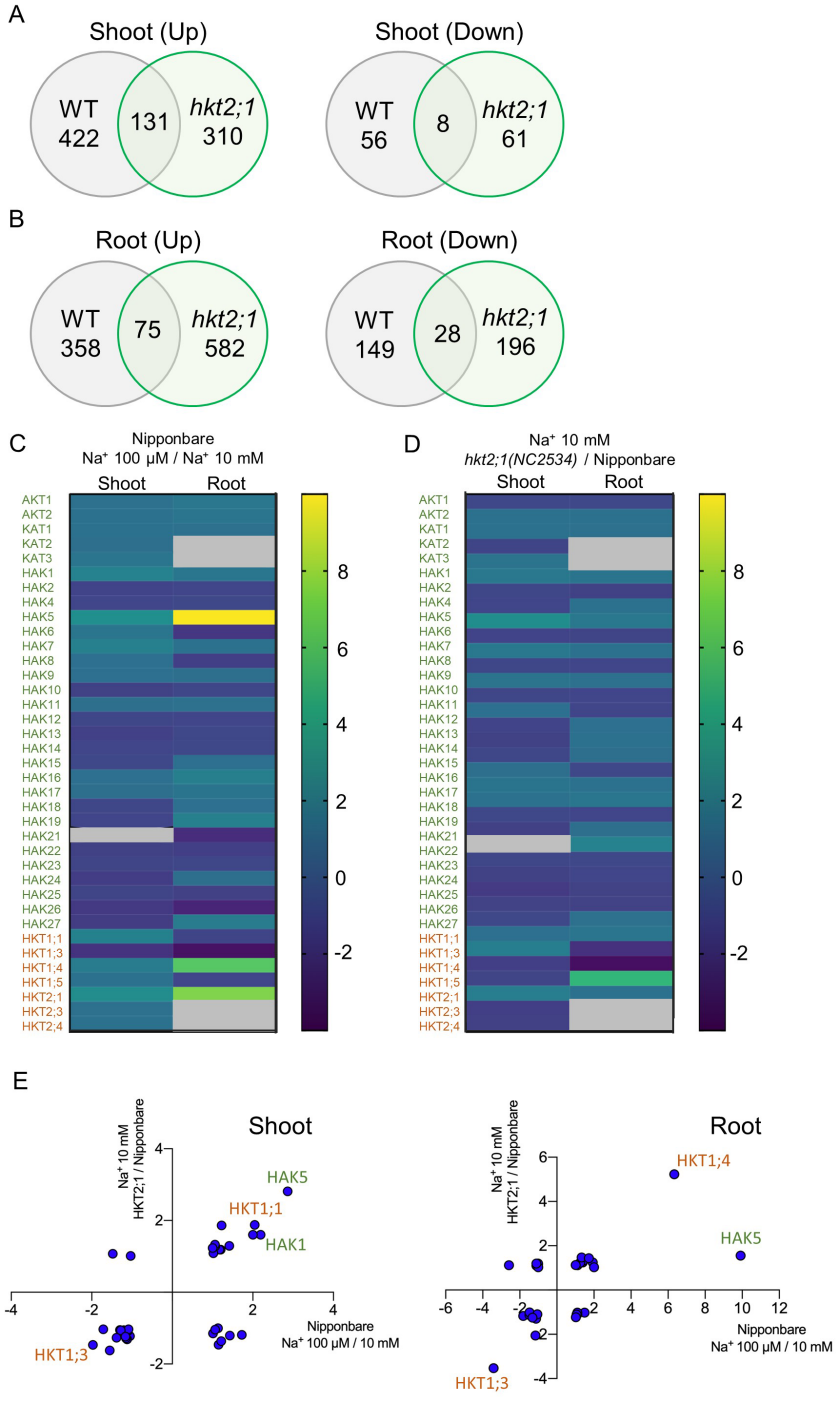
Expression differences of genes identified with RNA-Seq analysis. **(A)** Venn diagram of DEGs during increased Cs accumulation in shoot. Comparison of gene expressions that change when a high Cs concentration is observed in WT (Na^+^ 0.01 mM compared with 10 mM) and *hkt2;1* (*hkt2;1* in Na^+^ 10 mM compared with WT in Na^+^ 10 mM). DEGs were extracted for genes with fold change ≥ |2| and FDR *p* < 0.05. **(B)** Venn diagram of DEGs during increased Cs accumulation in root. Comparison is the same as **(A)**. **(C)** Comparison of K^+^ and Na^+^ transporter gene expressions of Nipponbare between 100 µM Na^+^ with high Cs^+^ translocation to and 10 mM Na^+^ with low Cs^+^ translocation. Green color shows K^+^ transporter, and orange color shows Na^+^ one. **(D)** Comparison of K^+^ and Na^+^ transporter gene expressions between *hkt2;1* with high Cs^+^ translocation and Nipponbare with low Cs^+^ translocation at Na^+^ 10 mM. Fold change was visualized with color bar and gray color in **(C)** and **(D)** panels means nonsignificant. **(E)** Correlation of genes that fluctuate in both conditions. Values of X and Y axes represent the fold change. Green color shows K^+^ transporter, and orange color shows Na^+^ one.

RNA sequencing (RNA-seq) analysis was performed to examine genes that change with shoot Cs accumulation. The gene expression profile after a 24-h treatment with 10 mM Na^+^ in WT was used as a reference to examine genes that changed in the 24-h 100 µM Na^+^ treatment of WT and 24 h 10 mM Na^+^ treatment of *hkt2;1*, both of which had higher Cs translocation from roots to shoots. There were 131 genes characterized by an increase in shoots and 8 genes characterized by a decrease in shoots (**Figure 5A**). In roots, 75 genes were commonly upregulated and 28 were commonly downregulated (**Figure 5B**). GO enrichment analysis was also performed on genes whose expression was commonly altered (**Supplementary Table 3**). Fold changes of K^+^ and Na^+^ transporters are indicated in **Figure 5C**. Differently expressed transporters between 100 µM Na^+^ (high shoot Cs) and 10 mM Na^+^ (low shoot Cs) are shown in **Figure 5C**, and those between *hkt2;1* (high shoot Cs) and Nipponbare (low shoot Cs) treated with 10 mM Na^+^ ae shown in **Figure 5D**. There was a commonality between WT and *hkt2;1* in the transporter genes that changed under high Cs translocation. Therefore, K^+^ and Na^+^ transporter variations were mapped separately in shoots and roots (**Figure 5E**). In shoots, *OsHAK5* and *OsHKT1;1*, were commonly upregulated by more than twofold. In roots, *OsHKT1;4* expression increased significantly and *OsHAK5* was well upregulated in WT but with less than twofold in *hkt2;1*. A common and clear decrease was found in *OsHKT1;3*, which decreased similar in shoot but less than half. There was an expectation of expression change in *OsHAK1*, which contributed to Cs**^+^** transport; however, the value varied approximately twofold in WT and less than twofold in *hkt2;1* (**Figure 5E**). Another Na**^+^** transport-related transporter gene, *NHXs*, which contributes to Na^+^ transport into the vacuole, and *SOSs*, which contributes to Na^+^ efflux out of the cell, did not vary under our experimental conditions (data not shown).

## 4 Discussion

### 4.1 Differences in Na contribution to Cs^+^ absorption at root and Cs^+^ translocation from roots to shoots

**Figure 6:**
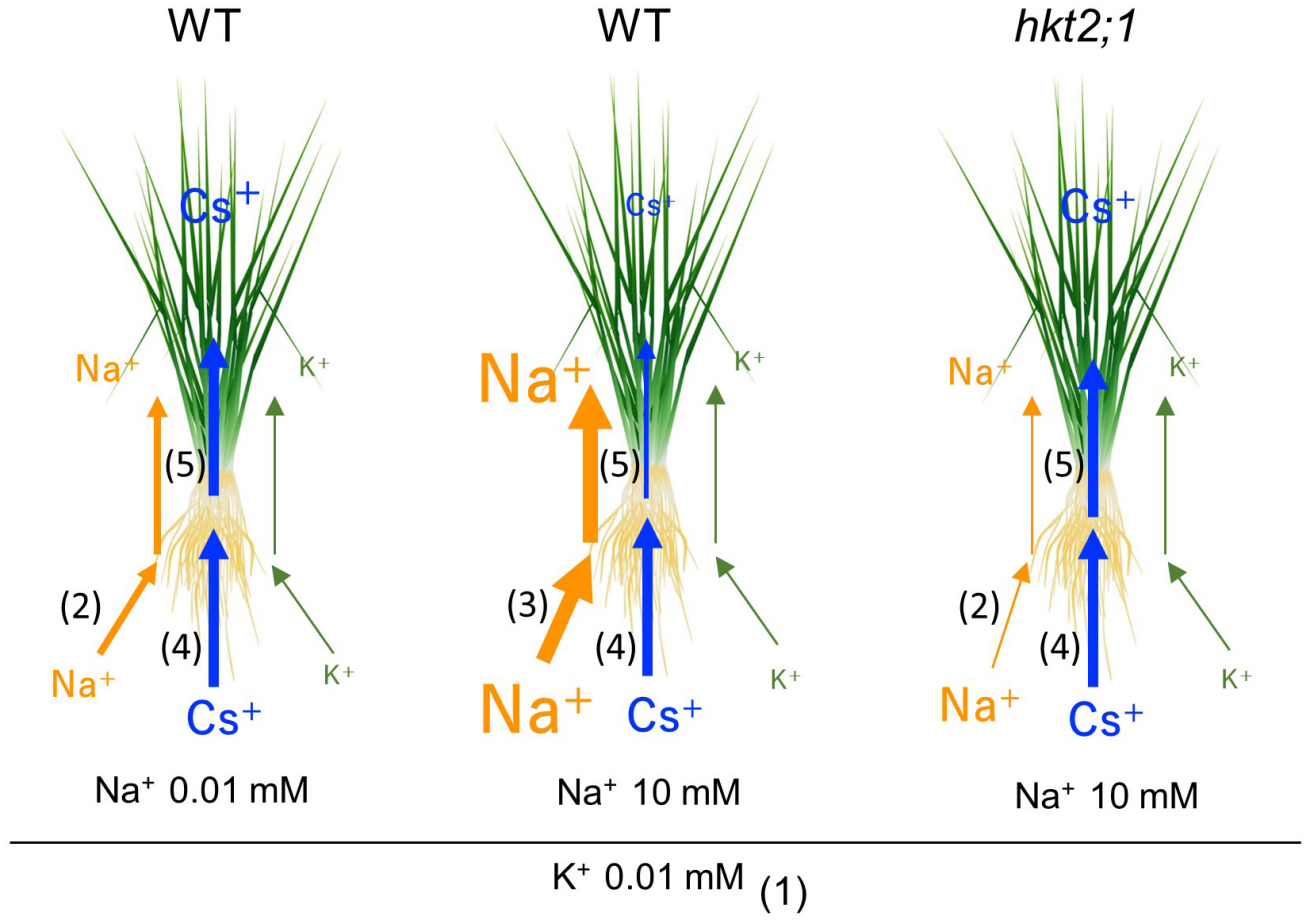
Effect of internal Na concentration on Cs translocation under low K conditions. Size of element indicates its concentration and width of arrow indicates the activity of transport. (1) Low K^+^ conditions increase the expression levels of K^+^ transporters, including *OsHKT2;1*. (2) Because of the K^+^ and Na^+^ co-transport activity of OsHKT2;1, alternative absorption of Na^+^ occurs. However, in *hkt2;1*, Na^+^ absorption is suppressed up to 10 mM Na^+^ condition. (3) If the Na^+^ concentration is high in the soil solution, Na^+^ absorption is enhanced in a concentration-dependent manner. (4) Under the low K^+^ conditions, Cs^+^ uptake also occurs. (5) However, Cs^+^ translocation from root to shoot indicates competing behavior with plant Na^+^ accumulation. Low Na^+^ accumulation arrows high Cs^+^ accumulation in shoot; however, high Na^+^ accumulation suppresses shoot Cs^+^ accumulation.

Previous studies reported the interrelationship between Na^+^ and Cs^+^ absorption in the mutant of the Na^+^ efflux system (Ishikawa et al., 2017). Ishikawa et al. isolated *lcs1* as a mutant in which Cs absorption was suppressed. The *lcs1* causative gene was *SOS2* responsible for the Na^+^ efflux system, called the SOS pathway. Under >5-mM Na^+^ conditions, the mutant with high Na^+^ concentration in the cytosol due to the loss of the Na^+^ efflux system suppressed Cs^+^ uptake via *OsHAK1* downregulation. This finding was similar to ours that Cs^+^ and Na^+^ transport is inversely related. However, under the previously reported conditions, the difference in Cs accumulation was observed in both the shoot and root and the decrease in shoot might be a subsequent effect of root Cs decrease, suggesting that the decrease in Cs translocation from root to shoot in this study is a new regulatory process of Cs distribution in plants. This was probably due to the preculture K^+^ conditions and plant K nutritional status. K^+^ conditions during the cultivation by Ishikawa et al. differed from our study, and the K^+^ concentration in the medium during the 5 days before the experiment was 200 µM and 20 times higher, whereas the Na^+^ concentration range was the same as our study. Probably under 200 µM K^+^ for 5 days condition, low but some level of K^+^ was present in the plant; therefore, short (2 h) Na^+^ treatment drastically suppressed *OsHAK1*, *OsHAK5*, and *OsHKT2;1* expression in root for inhibiting Na^+^ (and Cs^+^) uptake. Conversely, 10 µM K^+^ for 5 days might cause a severe K deficiency response, with insufficient K amount at root and shoot. This promoted K^+^-alternative Na^+^ and Cs^+^ absorption via OsHKT2;1 and OsHAK1, respectively (**Figure 4**; **Supplementary Figure 1B**). When available Na^+^ was low in the solution and internal Na^+^ level was not enough for K^+^ compensation, Cs^+^ translocation was further continued as another K^+^-alternative translocation mechanisms, as shown in a difference in the Cs amount in shoot in **Figure 4C**. These indicated that sufficient or insufficient K^+^ in the plant body alters the process of not only root absorption but root-to-shoot translocation of K^+^ alternative ions, and **Figure 6** summarizes our findings. Low K^+^ conditions increase the expression levels of K^+^ transporters, including *OsHKT2;1*. OsHKT2;1 have the K^+^ and Na^+^ co-transport activity; therefore, absorption of Na^+^ occurs in WT. However, in *hkt2;1*, Na^+^ absorption is suppressed up to 10 mM Na^+^ condition (**Figure 4A**). Under the low K^+^ concentration condition in the soil solution, the absorption of Cs^+^ was enhanced. However, Cs^+^ translocation from root to shoot indicates competing behavior with plant Na^+^ accumulation and high Na^+^ accumulation suppress shoot Cs^+^ accumulation (**Figures 4A, C**).

### 4.2 Contribution of OsHAKs to Cs^+^ transport in this study

In the previous studies, a K^+^ transporter OsHAK1 transports Cs^+^ and its expression level is enhanced under low K^+^ conditions. It was expected that the high Cs^+^ transport in *hkt2;1* might be due to OsHAK1. However, no significant difference was found in the expression level of *OsHAK1* during 24-h Na^+^ treatment which changed the shoot Cs concentration among WT and *hkt2;1*. On the other hand, comprehensive gene expression analysis results by RNA-seq showed that *OsHAK5*, *OsHKT1;3*, and *OsHKT1;4* genes were significantly affected (**Figure 5**). Since the beginning of this study, there has been high interest in *OsHAK5* because its gene expression increases under K deficiency (Yang et al., 2014). Therefore, field experiments on *hak5* and *hkt2;1* were both conducted, but the reduction in Cs accumulation due to *hak5* loss was observed in brown rice under K fertilized conditions (**Supplementary Figure 4**). In general, HAK5 function is necessary under low K conditions; therefore, the difference observed under K fertilized conditions is difficult to consider. A recent study using rice cells whose *OsHAK5* was knocked down by RNA interference also showed OsHAK5 greatly contributed to K^+^ absorption under limited K^+^ conditions but not in Cs^+^ (Uchiyama et al., 2022). Therefore, our findings related to Cs translocation from root to shoot should be controlled by other mechanisms and the identification of it will provide the novel knowledge about Cs^+^ transport in the plants.

### 4.3 Role of OsHKT1;3 and OsHKT1;4

To investigate the cause of increased Cs translocation due to low Na in the plant body, the expression levels of genes coding K^+^ and Na^+^ transporters were compared based on RNA-seq results (**Figure 5**): *OsHKT1;3* was downregulated in shoots and roots, whereas *OsHKT1;4* was upregulated in roots. Based on the negative relationship between shoot Cs and root Na concentrations (**Supplementary Figure 3**; **Supplementary Table 2**), the change of gene expression was mainly focused with root. Both class 1 HKTs had Na^+^ transport capacity. OsHKT1;4 showed strong Na^+^ selectivity among cations tested, including Li^+^, Na^+^, K^+^, Rb^+^, Cs^+^, and NH_4_^+^, in oocytes (Suzuki et al., 2016b). Under 25-mM to 100-mM Na^+^ conditions, OsHKT1;4 was involved in Na^+^ exclusion in stems and leaf sheaths of xylem, thus excluding Na^+^ from leaf blades (Suzuki et al., 2016b). However, OsHKT1;4 was downregulated in roots in Na stressed conditions in our experimental condition. In low Na^+^ conditions (0.5 mM–5 mM), Khan et al. found that the transporter, expressed in *Xenopus* oocytes, displayed a relatively high affinity for Na^+^ and also progressive desalinization of the xylem sap Na^+^ concentrations in roots (Khan et al., 2020). Based on the GUS reporter assay, *OsHKT1;4* was mainly expressed in xylem parenchyma in roots and leaves. Even in experimental conditions, OsHKT1;4 may contribute to Na^+^ recovery from xylem. However, it is a puzzle why this function is constant even when Na^+^ in the xylem is low. The details of the transport function in roots and the Cs^+^ transport of OsHKT1;4 itself must be verified. OsHKT1;3 did not show any type of transport activity in yeast cells but mediated inward and outward Na^+^ currents in *Xenopus* oocytes (Rosas-Santiago et al., 2015; Garciadeblás et al., 2003). *OsHKT1;3* have been reported in five mRNA variants (Imran et al., 2021). Imran et al. analyzed functional differences between variants and found Na^+^ transport permeability. Because variants have different Na^+^ transport functions, they are expected to have different permeability to other cations. A detailed verification in monovalent cation transport activities in OsHKT1;3 and OsHKT1;4 should provide a new insight for specificity of ion selectivity of plant transporters.

### 4.4 Can Na addition be a method to inhibit Cs accumulation like K fertilization?

To control the transfer of radioactive materials to crops, increased application of K fertilizers has been continued in areas affected by the nuclear power plant accident. However, because the long-term application of large K amounts is costly and labor-intensive, there is a need for a technology that can reliably control the transfer of Cs while reducing the additional K application. Although Na is not an essential element for rice, it is recognized as a K^+^ replaceable element, and at low concentrations, Na does not cause growth disturbance (Horie et al., 2007; Ochiai et al., 2022). Rather, there is an alternative absorption of Na^+^ in rice at low K^+^, which contributes to keeping the rice biomass. This experiment showed that the moderate presence of Na^+^ suppresses the Cs content in the plant shoot and the results can hopefully be applied to an indicator of fertilizer that focuses on Na^+^ and K^+^ rather than only K^+^ in the fields.

This study indicated that the Na concentration within the root is negatively correlated with the Cs accumulation in shoots (**Figure 4**; **Supplementary Figure 3**). Genes regulated by low levels of Na might affect Cs^+^ translocation activity from roots to shoots (**Figure 5**). It is necessary to investigate in detail whether these candidate transporters could transport Cs^+^. Clarifying the importance of K^+^ and Na^+^ conditions in the Cs^+^ translocation mechanism would lead to understanding basic knowledge linked to the new applications such as improving soil environment for crop production not only by K^+^ but also by Na^+^.

## Data Availability

The original contributions presented in the study are publicly available. This data can be found here: DDBJ, PRJDB18915
