# Supplementary material for: Rice Na+ absorption mediated by OsHKT2;1 affected Cs+ translocation from root to shoot under low K+ environments
Source: Front Plant Sci. 2024 Oct 15;15:1477223. doi: 10.3389/fpls.2024.1477223 (PMC11518805; doi:10.3389/fpls.2024.1477223)
Supplement: Supplementary file 1 [file DataSheet1.pdf]

Supplementary Material

1. Supplementary Figures

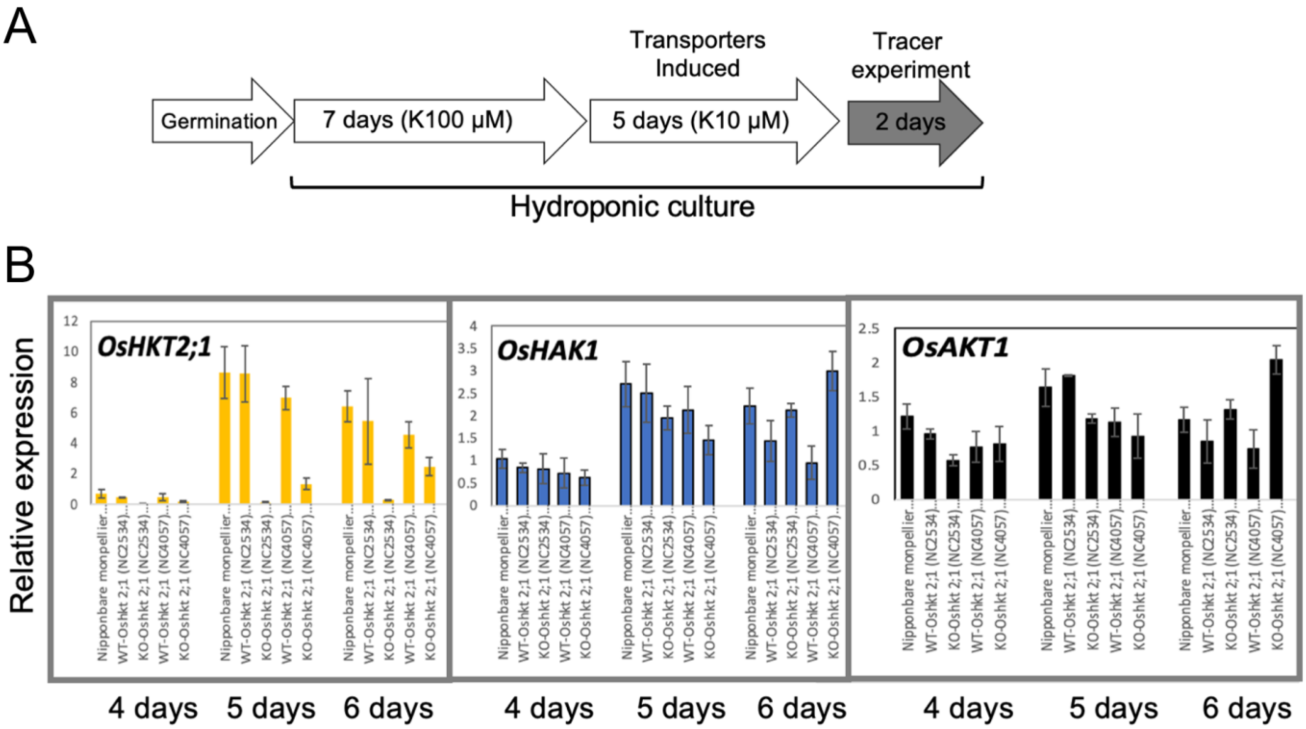

Supplementary Figure 1. (A). Plant growing conditions for investigating Na^{+} effect on ^{22}Na, ^{43}K and ^{137}Cs absorption. (**B)**. RT-qPCR analysis of K^{+} transporter gene expressions under 10 μM K^{+} treatment from day 4 to day 6 after 7 days cultivation with 100 μM K^{+}. Based on this gene expression results, samples were subjected to stress treatment for 5 days. Data are the mean ± SD (n = 4).

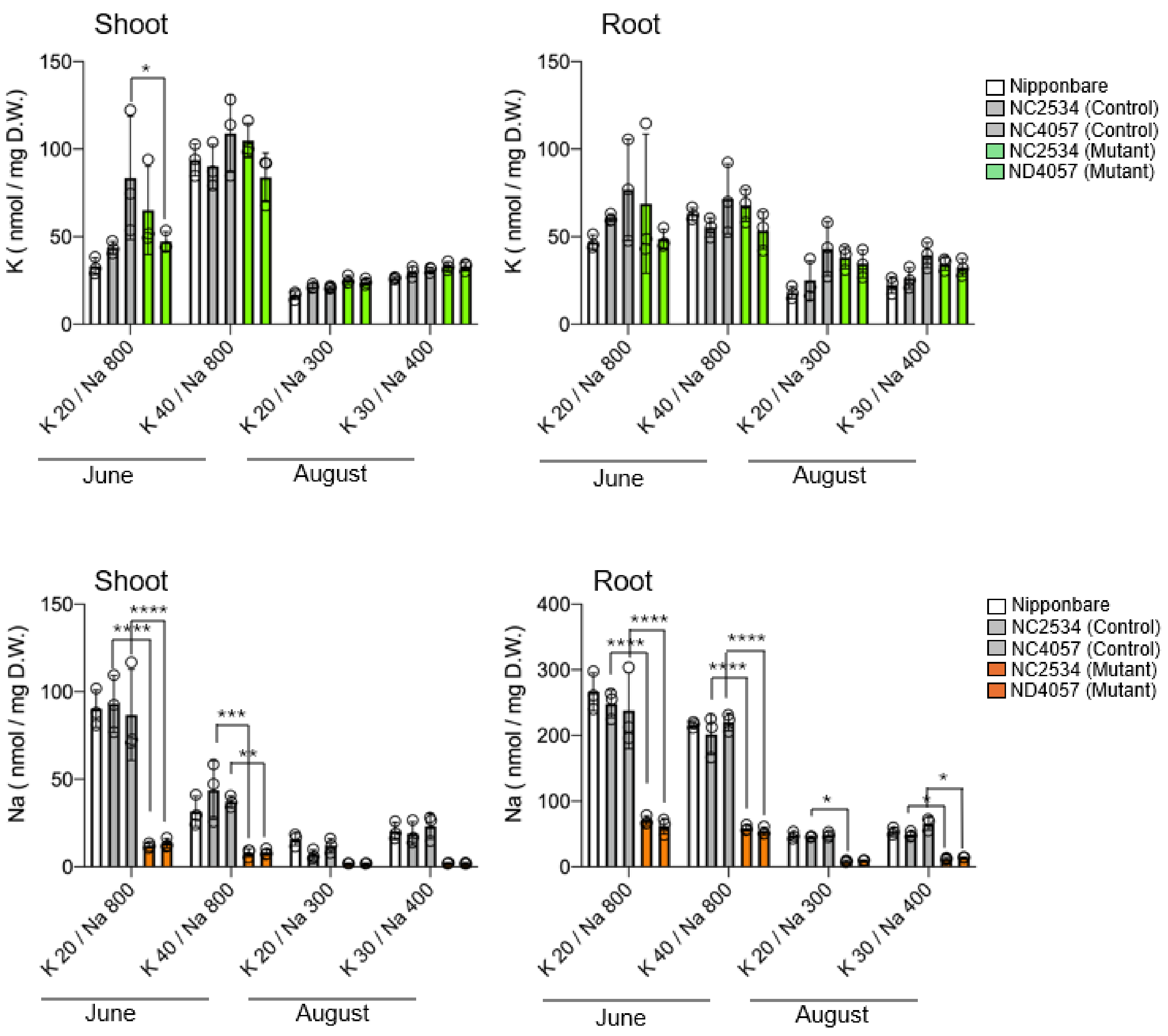

Supplementary Figure 2. K and Na absorption with hydroponic solutions reproducing Na^+ and K^+ concentrations in the paddy field. From left to right: Nipponbare (white), WT of NC2534, WT of ND4057 (gray), mutant type NC2534, and mutant type ND4057 (green for K and orange for Na). Data are the mean ± SD. n = 3 (biologically independent experiments) and individual data points as overlays. *p < 0.05, **p = 0.0098, ***p = 0.0005, ****p < 0.0001 (Two-way Anova, Tukey's multiple comparisons)

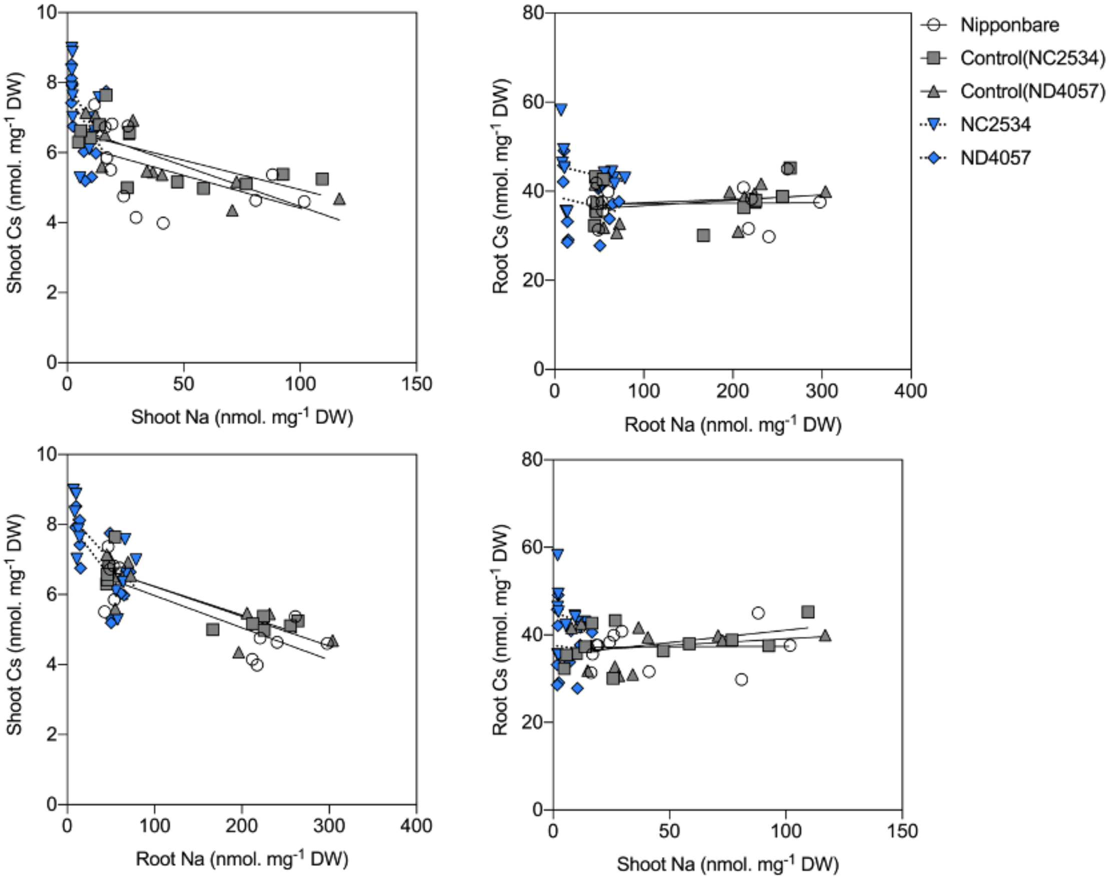

Supplementary Figure 3. Correlation between Cs and Na in an experimental hydroponic system. Negative correlation between Na and Cs accumulation were confirmed based on data from Figure 3 and Supplementary Figure 2.

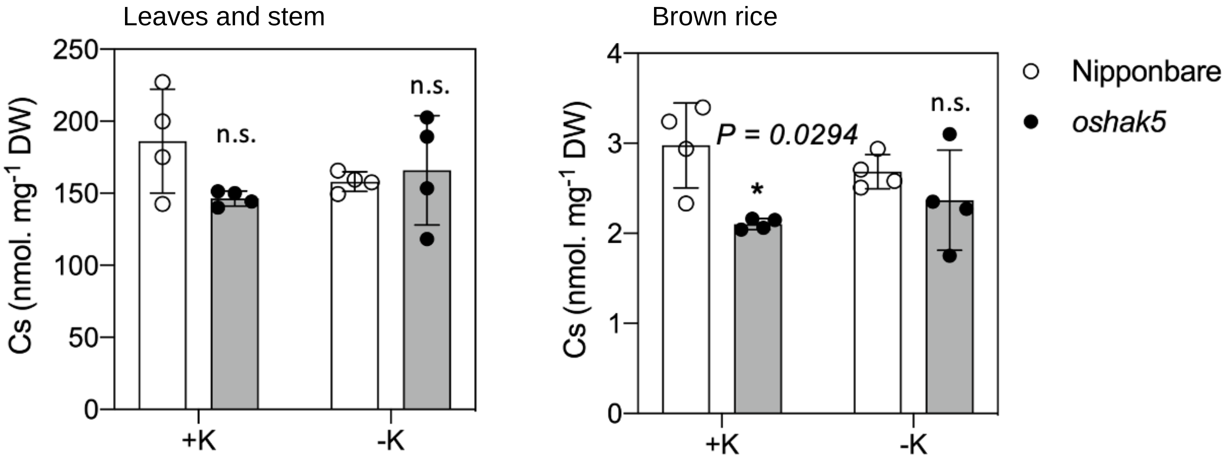

Supplementary Figure 4. ^{137}Cs accumulation test with *oshak5* in the paddy field. ^{137}Cs content of shoot samples grown for ~5 months in the ^{137}Cs-contaminated paddy field. Nipponbare as control (white) and mutant line of *OsHAK5* gene with Tos-17 insertion (gray). Data are the mean ± SD. n = 3 (biologically independent experiments) and individual data points as overlays. t-test with Welch's correction *$p < 0.05$. n.s. means not significant.

2. Supplementary Tables

Supplementary Table 1. Primer list for Supplementary Figure 1.

Primer name	Primer sequence 5’- 3’
Actin_Forward	GCGTGGACAAAGTTTTCAACCG
Actin_Reverse	TCTGGTACCCTCATCAGGCATC
Ubiquitin_Forward	CAACCAGCAGGCTTAGGCGTAG
Ubiquitin_Reverse	GGTGTTCAGTTCCAAGGAGAC
OsHAK1_Forward	GTTGATGATGCTGATGTTGGAAGAG
OsHAK1_Reverse	TTACACAAACCACACTGTACAGAG
OsAKT1_Forward	CAGTAATGAATGGGATGCAGAG
OsAKT1_Reverse	CCCCTTTCTTGGAAATCAACAG
OsHKT2;1 _Forward	TCCATCGACTGCTCACTCA
OsHKT2;1 _Reverse	TGTTGTCGATGGTGGTAAGTACA

Supplementary Table 2. Negative correlation between Na and Cs accumulation in wild type and *hkt2;1*.

Shoot Cs vs Shoot Na	Equation	R^2	P value
Nipponbare	Y = -0.01866X + 6.283	0.2649	0.0869
Control (NC 2534)	Y = -0.01676X + 6.619	0.4578	0.0157
Control (ND 4057)	Y = -0.02314X + 6.778	0.6117	0.0026
NC 2534	Y = -0.1268X + 8.076	0.2635	0.0879
NC 4057	Y = -0.1034X + 7.637	0.2255	c0.1188

Shoot Cs vs Root Na	Equation	R^2	P value
Nipponbare	Y = -0.009192X + 6.888	0.6609	0.0013
Control (NC 2534)	Y = -0.008180X + 7.051	0.7662	0.0002
Control (ND 4057)	Y = -0.008475X + 7.071	0.7028	0.0007
NC 2534	Y = -0.02726X + 8.334	0.4912	0.0111
NC 4057	Y = -0.03318X + 8.134	0.5193	0.0082

Simple linear regression was calculated from Supplementary Figure 3.

Supplementary Table 3. GO analysis of Figure 5.

A. A group of genes commonly upregulated in shoot

GO term	GO accession	Number of gene	p value
cytoplasmic part	GO:0044444	63	5.1E-61
cytoplasm	GO:0005737	65	4.7E-57
intracellular membrane-bounded organelle	GO:0043231	71	6.1E-49
membrane-bounded organelle	GO:0043227	71	1.2E-48
intracellular organelle	GO:0043229	71	1.3E-42
organelle	GO:0043226	71	1.3E-42
cytoplasmic membrane-bounded vesicle	GO:0016023	22	7.7E-41
membrane-bounded vesicle	GO:0031988	22	7.7E-41
vesicle	GO:0031982	22	5.9E-39
cytoplasmic vesicle	GO:0031410	22	5.9E-39
intracellular part	GO:0044424	73	6.7E-39
intracellular	GO:0005622	75	1.2E-36
plastid	GO:0009536	21	2.8E-36
cell part	GO:0044464	80	7.2E-31
cell	GO:0005623	80	7.2E-31
mitochondrion	GO:0005739	20	3.7E-23
oxidoreductase activity, acting on single donors with incorporation of molecular oxygen, incorporation of two atoms of oxygen	GO:0016702	6	1.3E-08
dioxygenase activity	GO:0051213	6	2.3E-08
oxidoreductase activity, acting on single donors with incorporation of molecular oxygen	GO:0016701	6	5.9E-08
lyase activity	GO:0016829	9	7E-06
carboxylic acid metabolic process	GO:0019752	10	3.2E-05
oxoacid metabolic process	GO:0043436	10	3.2E-05
organic acid metabolic process	GO:0006082	10	3.3E-05
cellular ketone metabolic process	GO:0042180	10	3.6E-05
regulation of transcription, DNA-dependent	GO:0006355	11	0.00027
regulation of RNA metabolic process	GO:0051252	11	0.00028
transcription, DNA-dependent	GO:0006351	11	0.00046

B. A group of genes commonly upregulated in root.

GO term	GO accession	Number of gene	p value
cytoplasmic part	GO:0044444	76	4E-72
cytoplasm	GO:0005737	80	1E-69
vesicle	GO:0031982	32	3E-57
cytoplasmic vesicle	GO:0031410	32	3E-57
intracellular membrane-bounded organelle	GO:0043231	80	2.8E-50
membrane-bounded organelle	GO:0043227	80	5.8E-50
intracellular organelle	GO:0043229	81	2.1E-44
organelle	GO:0043226	81	2.1E-44
intracellular part	GO:0044424	85	6E-42
intracellular	GO:0005622	87	6.9E-39
plastid	GO:0009536	23	4E-38
cell part	GO:0044464	96	4.6E-35
cell	GO:0005623	96	4.6E-35
mitochondrion	GO:0005739	19	7.6E-20
terpenoid metabolic process	GO:0006721	5	2.5E-08
isoprenoid metabolic process	GO:0006720	5	0.0000036
secondary metabolic process	GO:0019748	5	0.000027

C. A group of genes commonly downregulated in root.

GO term	GO accession	Number of gene	p value
cytoplasmic membrane-bounded vesicle	GO:0016023	10	2.6E-24
membrane-bounded vesicle	GO:0031988	10	2.6E-24
vesicle	GO:0031982	10	2.5E-23
cytoplasmic vesicle	GO:0031410	10	2.5E-23
cytoplasmic part	GO:0044444	11	5.5E-11
cytoplasm	GO:0005737	11	9.3E-10
intracellular membrane-bounded organelle	GO:0043231	11	0.00000045
membrane-bounded organelle	GO:0043227	11	0.0000005
cell part	GO:0044464	15	0.0000033
cell	GO:0005623	15	0.0000033
intracellular organelle	GO:0043229	11	0.000004
organelle	GO:0043226	11	0.000004
response to chemical stimulus	GO:0042221	5	0.0000042
intracellular part	GO:0044424	11	0.000031
response to stress	GO:0006950	6	0.000058
intracellular	GO:0005622	11	0.00013
response to stimulus	GO:0050896	6	0.00014
membrane	GO:0016020	8	0.00017

GO enrichment analysis was performed by AgriGO for genes that commonly fluctuate between WT and *hkt2;1*. (A) Group of genes commonly upregulated in shoots, (B) group of genes commonly upregulated in roots, and (C) group of genes commonly downregulated in shoots. GO enrichment analysis was not possible because there were a few genes whose gene expression was downregulated in shoots.
